# What a stranded whale with scoliosis can teach us about human idiopathic scoliosis

**DOI:** 10.1038/s41598-021-86709-x

**Published:** 2021-03-30

**Authors:** Steven de Reuver, Lonneke L. IJsseldijk, Jelle F. Homans, Dorien S. Willems, Stefanie Veraa, Marijn van Stralen, Marja J. L. Kik, Moyo C. Kruyt, Andrea Gröne, René M. Castelein

**Affiliations:** 1 Department of Orthopaedic Surgery, University Medical Center Utrecht, Utrecht, The Netherlands; 2 Division of Pathology, Department of Biomolecular Health Sciences, Faculty of Veterinary Medicine, Utrecht University, Utrecht, The Netherlands; 3 Division of Diagnostic Imaging, Department of Clinical Sciences, Faculty of Veterinary Medicine, Utrecht University, Utrecht, The Netherlands; 4 Imaging Division, University Medical Center Utrecht, Utrecht, The Netherlands

**Keywords:** Biophysics, Evolution, Zoology, Anatomy, Diseases, Medical research, Pathogenesis

## Abstract

Scoliosis is a deformation of the spine that may have several known causes, but humans are the only mammal known to develop scoliosis without any obvious underlying cause. This is called ‘idiopathic’ scoliosis and is the most common type. Recent observations showed that human scoliosis, regardless of its cause, has a relatively uniform three-dimensional anatomy. We hypothesize that scoliosis is a universal compensatory mechanism of the spine, independent of cause and/or species. We had the opportunity to study the rare occurrence of scoliosis in a whale (*Balaenoptera acutorostrata*) that stranded in July 2019 in the Netherlands. A multidisciplinary team of biologists, pathologists, veterinarians, taxidermists, radiologists and orthopaedic surgeons conducted necropsy and imaging analysis. Blunt traumatic injury to two vertebrae caused an acute lateral deviation of the spine, which had initiated the development of compensatory curves in regions of the spine without anatomical abnormalities. Three-dimensional analysis of these compensatory curves showed strong resemblance with different types of human scoliosis, amongst which idiopathic. This suggests that any decompensation of spinal equilibrium can lead to a rather uniform response. The unique biomechanics of the upright human spine, with significantly decreased rotational stability, may explain why only in humans this mechanism can be induced relatively easily, without an obvious cause, and is therefore still called ‘idiopathic’.

## Introduction

Scoliosis is a three-dimensional (3D) deformity of the spine and trunk, in which rotation of the vertebral column in the horizontal plane together with extension in the sagittal plane plays a consistent role, that may be caused by traumatic injury, syndromic conditions, congenital malformations or neuromuscular disease^1^. In mammals, the development of scoliosis without an obvious underlying cause is exclusively observed in humans, this is called ‘idiopathic’ scoliosis and is the most frequently observed type^1–3^. The condition occurs with a prevalence of 1–4% in otherwise healthy individuals, most commonly adolescent females^1^. Treatment is currently focused on limiting progression of the spinal curve until skeletal maturity, which can necessitate bracing therapy or spinal fusion surgery^1^. Many theories have been brought forward in search of the aetiology of idiopathic scoliosis^1,4–12^. Upright spinal biomechanics, that implies a reduction of stability in the horizontal plane, was shown to play an important role^13–19^. And while the shape of the scoliotic spine has been described for over a century^10,20, 21^, recent observations have shown that the 3D anatomy is very uniform across human scoliosis with different aetiology, including vertebral rotation into the curve convexity and anterior lengthening of the intervertebral discs^22–26^.

We hypothesize that scoliosis is a universal compensatory mechanism of the spine, that consists of vertebral rotation into the convexity of the curve, accompanied by anterior lengthening of the intervertebral discs, that can be caused by different primary challenges to spinal equilibrium. One of these challenges, and a possible explanation of idiopathic scoliosis in humans is the unique upright posture with the centre of weight balanced straight above the pelvis, resulting in a unique biomechanical loading of the trunk^13–17,27^. This specific sagittal plane configuration of the human spine was shown to lead to decreased rotational stability, making it more prone than other spines in nature to decompensate into scoliosis^18,19,28,29^. Scoliosis is found rarely in other vertebrates than humans and is usually caused by anatomic abnormalities^30,31^.

**Figure 1:**
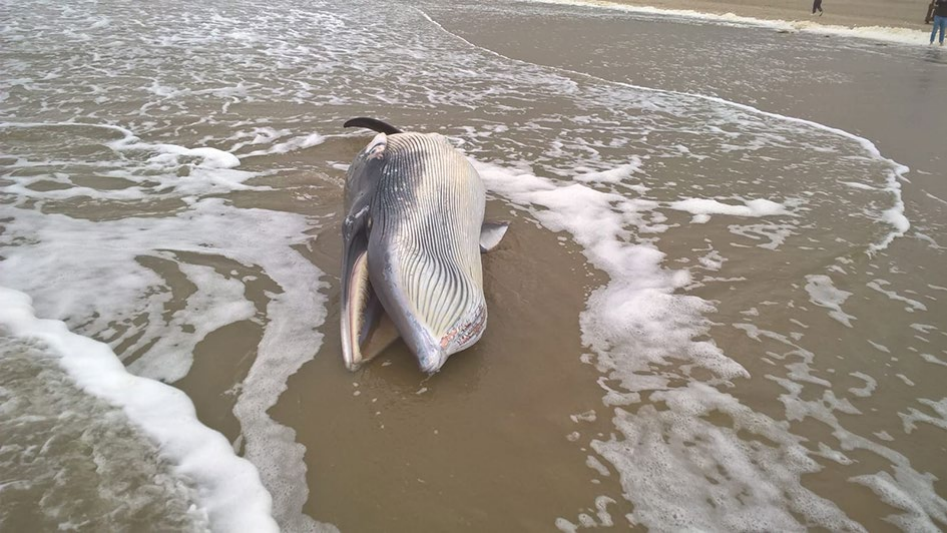
Photograph of the common minke whale (*Balaenoptera acutorostrata*) that washed ashore on the 8th of July 2019 at Texel, the Netherlands. Photograph by Pierre Bonnet (Ecomare, Texel).

Recently, we had the opportunity to study the *compensatory curves* in an anatomically normal area of the spine of a whale with a post-traumatic scoliosis. Whales are sea mammals that are not known to develop scoliosis spontaneously: several reports on cetaceans with scoliosis exist, however all cases have a clear cause which is mostly of traumatic origin, e.g. following ship collision^32–34^. In the current study we examined a young common minke whale (*Balaenoptera acutorostrata*), which was found stranded in July 2019 in the Netherlands with an obvious spine trauma and subsequent severe local post-traumatic scoliosis (Fig. 1). This post-traumatic scoliosis initiated compensatory 3D curves in the area of the spine that was not affected by the trauma, apparently in an attempt of the animal to re-align its trunk. We were interested in these compensatory curves, as they could provide insights into the more general, intrinsic mechanisms that govern alignment of the mammalian spine. A multidisciplinary team of biologists, pathologists, veterinarians, taxidermists, radiologists and orthopaedic surgeons studied the whale and conducted a necropsy and 3D imaging analysis of the spine and compared the findings to non-scoliotic whales. The aim of the study was to assess whether scoliosis is a universal compensatory mechanism that occurs independent of cause and/or species. The hypothesis tested in the current study was that the injured whale would re-align its trunk by creating compensatory curves in the essentially normal spine and that these curves show a similar 3D configuration as is observed in human scoliosis.

## Results

### Post mortem findings

The common minke whale was a 403 cm long, 530 kg female juvenile, with an estimated age between 0.5 and 4 years^35^. Besides the clear lateral post-traumatic curvature of the spine, other important findings of external examination were multifocal areas of deep haemorrhage and oedema that were present in the subcutis and longissimus dorsi muscle, as well as the presence of blood tinge liquid in the spinal canal and congestion and haemorrhage of the brain. The animal had a poor nutritional condition (blubber layers of 20–25 mm) despite recent feeding^36^. Histology of the fractured vertebrae demonstrated fibrin deposits, some eosinophilic granulocytes, and necrosis, indicative of chronic changes that were still ongoing. Therefore, the most likely cause of death was considered to be acute recent blunt trauma.

**Figure 2:**
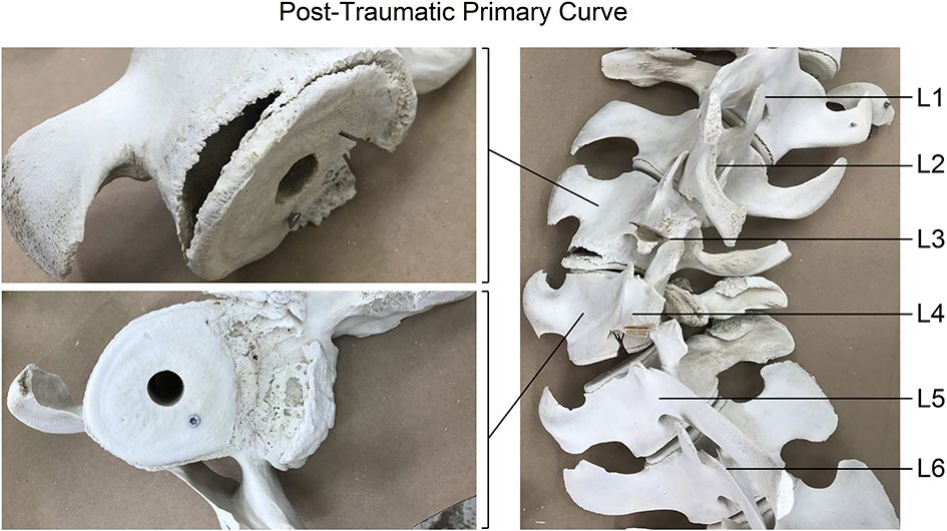
Photographs of the post-traumatic primary curve after removing the soft tissues. The dorsal overview on the right-hand side shows the post-traumatic primary, abrupt coronal curve at level L3/L4. Close-up inspection reveals an epiphysiolysis at the left-side of the lower endplate of vertebra L3, and a burst upper endplate at the right-side of vertebra L4. Furthermore, fractured spinous processes at multiple levels are present. There are multiple post mortem marks following tissue selection for histopathology, and also centre holes and screws that were drilled through the endplates in the process of framing the complete skeleton for museum display. These artefacts did not influence the presented post-traumatic features.

Furthermore, there was clear evidence that earlier trauma had resulted in the fractures and other deformations of the lumbar vertebrae, which had led to a localized, post-traumatic deformity of the spine. Visual inspection showed that the deformity was mostly in the coronal plane with no significant lordosis or kyphosis at that region. This was further investigated after removing all of the soft tissues of the entire vertebral column. Visual inspection showed an epiphysiolysis at the left-side of the lower endplate of vertebra L3, a burst upper endplate at the right-side of vertebra L4 and fractured/missing spinous processes at multiple levels (Fig. 2).

**Figure 3:**
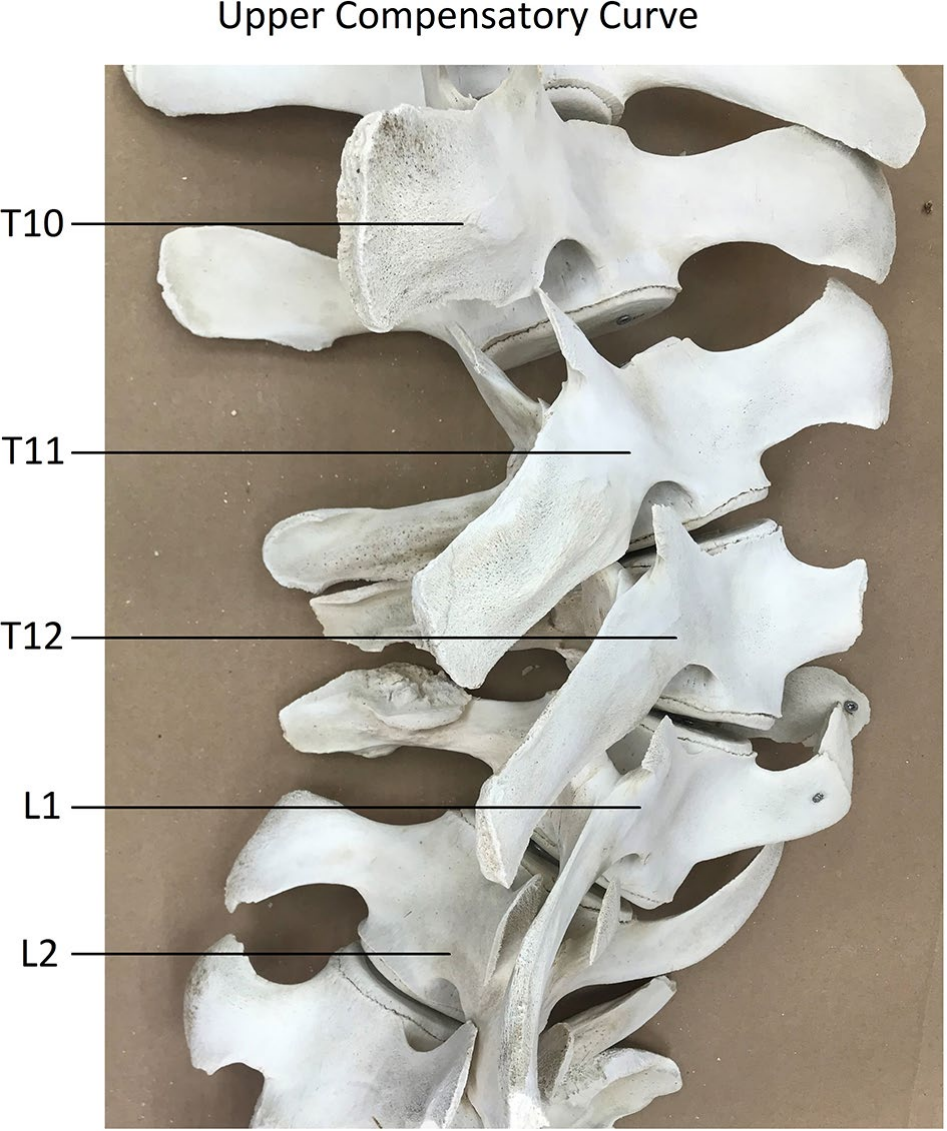
Close-up photograph of the apex of the upper compensatory curve, directly cranial of the post-traumatic primary curve, after removing the soft tissues. This compensatory curvature occurred in an (initially) anatomically normal part of the spine, unaffected by the trauma.

**Figure 4:**
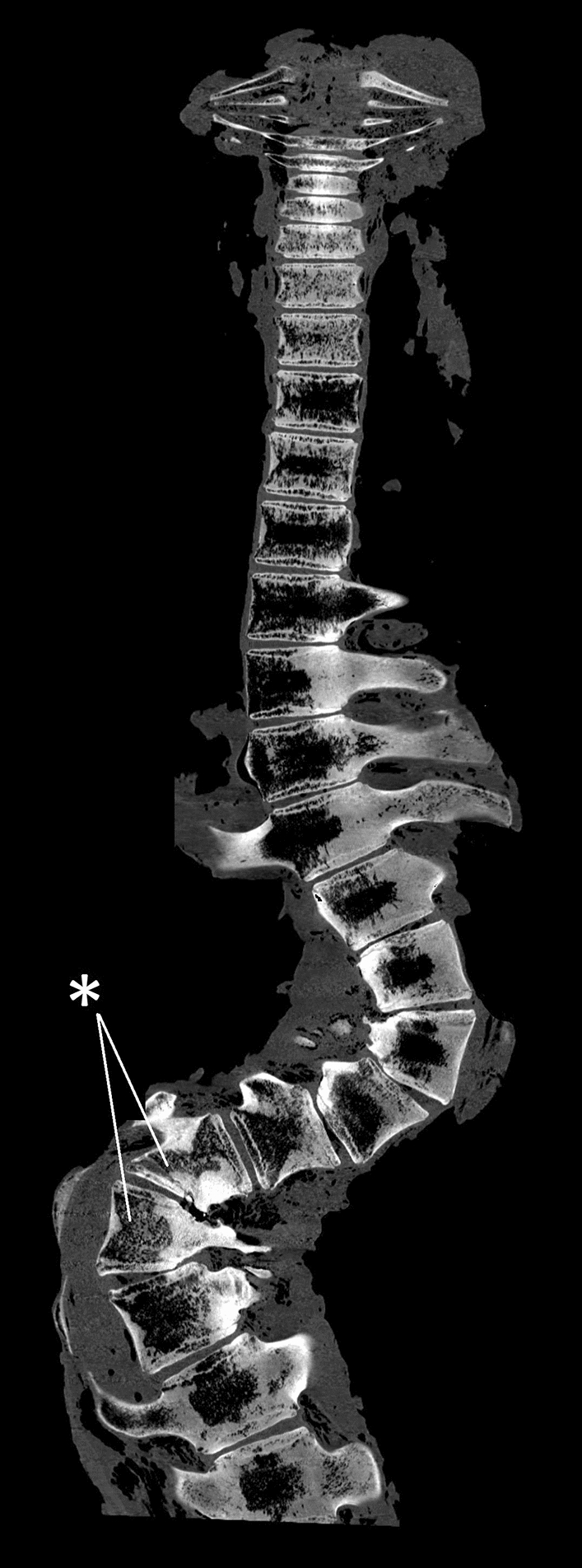
Dorsal view with the cranial side upwards from the CT-scan of level C1 to L7. The site of the blunt traumatic injury at level L3/L4 (indicated with an asterisk) initiated a double compensatory curve cranially and a single compensatory curve caudally.

Furthermore, spinal curvatures were observed in the adjacent, anatomically normal parts of the spine, outside the traumatically affected area (Fig. 3). Therefore, the thoraco-lumbar area was the suspected site of an acute (dorso-)lateral blunt traumatic injury, which subsequently initiated a double compensatory curve cranially and a single compensatory curve caudally in areas of the spine unaffected by the trauma (Fig. 4). We analysed these compensatory curves in 3D and compared the morphology with the non-scoliotic spine of 10 control whales. The levels T11/12 (severe wedging) and L3/L4 (traumatic injury) were excluded before CT-scan analysis of the compensatory curvatures.

### CT-measurements

**Figure 5:**
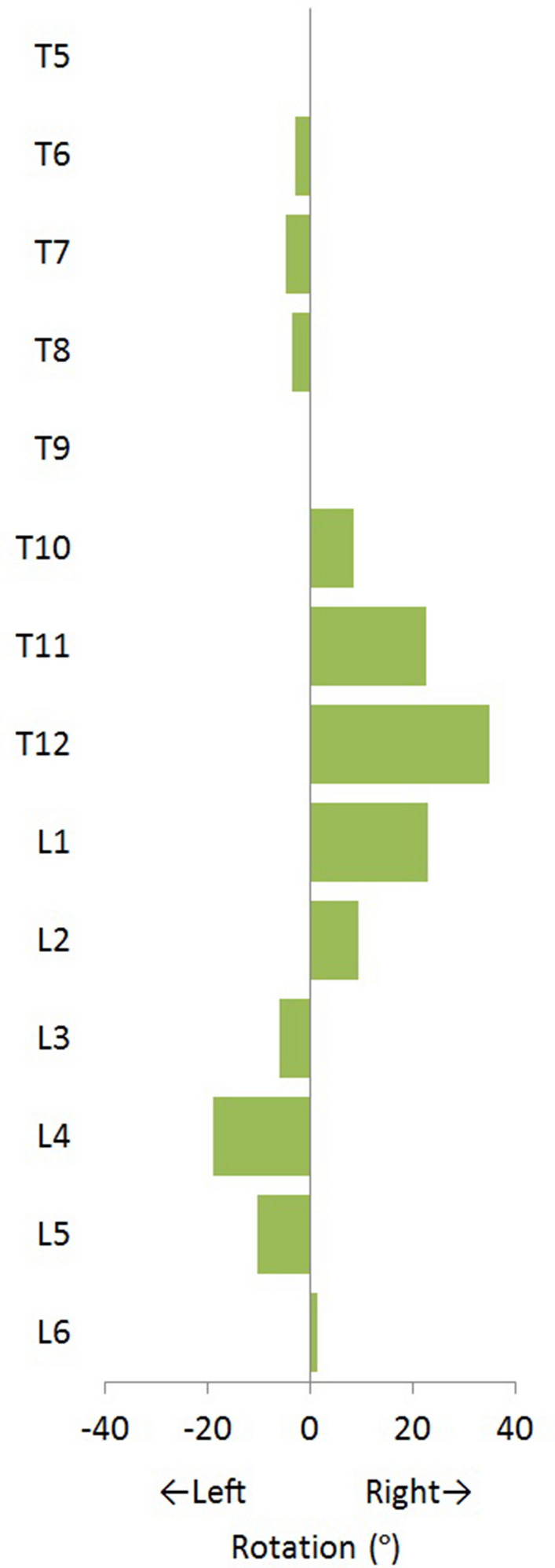
Of the whale with scoliosis, the rotation of the vertebral bodies in the transverse plane is shown in degrees. Positive values indicate that the anterior part of the vertebral body is pointing towards the right. All rotation of the vertebral bodies is into the convexity of the curve.

**Figure 6:**
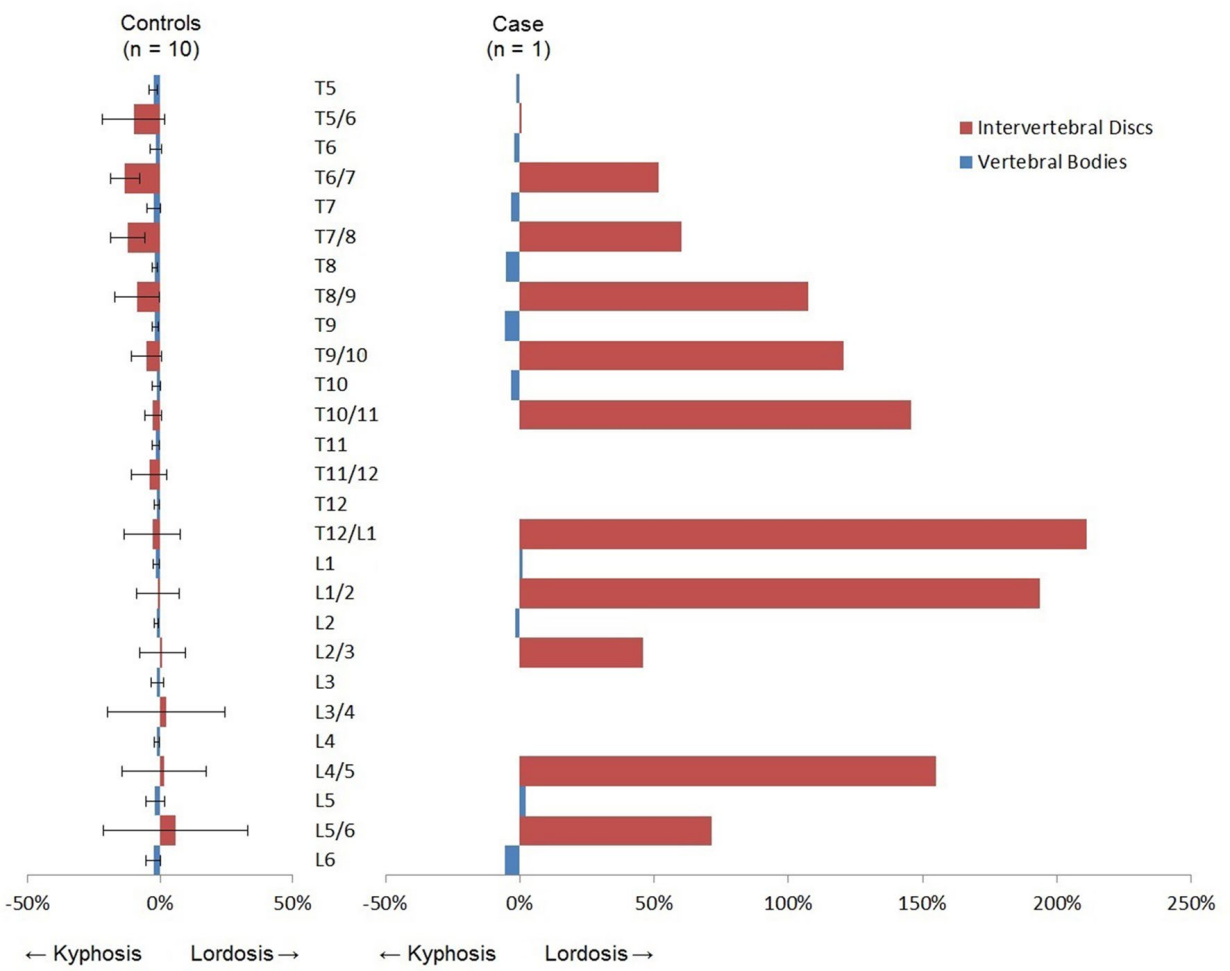
The mean anterior–posterior length discrepancy (AP%) with the standard deviation is shown for the intervertebral discs in red and the vertebral bodies in blue, for both the non-scoliotic controls and the whale with scoliosis. Endplates severely affected by trauma (L3/L4) and wedging (T11/T12) were excluded due to impossibility of proper CT-scan analysis. Positive AP% indicates a larger anterior length than posterior length.

The 3D analysis of the compensatory curves showed a rotation of the vertebral bodies in the transverse plane into the convexity of the curve (Fig. 5). The mean anterior–posterior length discrepancy (AP%) of the total compensatory curvature was + 9.4% in the whale. This means that the anterior (ventral) length of the compensatory scoliotic curvature was 9.4% greater than the posterior (dorsal) length, indicating a regional lordosis. This is significantly different from the kyphosis in the same part of the spine in the non-scoliotic control group, with a total AP% of − 2.1 ± 0.4%, meaning that the anterior length of the spine was 2.1% shorter than the posterior length (p < 0.001). On the contrary, the bony morphology of the vertebral bodies was similar to the controls; the vertebral body AP% of the whale was − 2.5%, which was comparable to the kyphotic shape of the vertebral bodies in controls with − 1.8 ± 0.8% (p = 0.429). Almost all anterior lengthening took place in the intervertebral discs, as the disc AP% in the compensatory curvature of the whale was + 99.5%, which meant a lordotic shape of the intervertebral discs with an anterior length almost twice the posterior length. This is in sharp contrast to the kyphosis in the discs of controls with − 4.6 ± 5.0% (p < 0.001). The AP% for the separate vertebral bodies and intervertebral discs at every level is shown in Fig. 6.

## Discussion

Idiopathic scoliosis is a 3D decompensation of a spine with no anatomical abnormalities, in an individual without underlying manifest disease^1^. In the search for its aetiology, many theories involving just as many of the body’s organ systems have been suggested to play a role^1,4–12^. The usually present lengthening of the anterior side of the thoracic spine in idiopathic scoliosis^5,6,37^, was suggested to be the result of a generalized bony overgrowth disorder (relative anterior spinal overgrowth; RASO), possibly as a compensation for a disturbance of synchronized growth between the neural and the osseous elements^7–9,38^. Recent observations have shown that this anterior lengthening occurs predominantly in the discs and is not restricted to idiopathic scoliosis^22–26^.

We propose that scoliosis is a rather universal compensatory mechanism that can occur as a response to a (perceived) disturbance of spinal equilibrium. The crucial difference between the human spine and that of other mammals is not in its anatomy, but in the way it is biomechanically loaded, not by the fact that man is bipedal (many species are) but by the fact that humans carry their center of mass more posteriorly than any other species^13–17,27^. This leads to a sagittal profile that makes the human spine, in comparison with any other spine in nature, quadrupedal and bipedal alike, a rotationally less stable structure^18,19,39^. This means that, whereas in other species often draconic measures are necessary to induce a scoliosis^3^, in humans much less is needed to initiate this mechanism. We propose that the possible value of scoliosis research in experimental animals is not in the primary, artificially induced curve, but in the response that follows in the untouched area of the spine, i.e. the compensatory curve. This is supported by the observation that compensatory curves in congenital scoliosis in humans show a very similar 3D morphology to idiopathic scoliosis^25^, and that in porcine tether induced scoliosis the compensatory curves outside the instrumented spinal segment showed a similar rotational deformity^40^.

The objective of the current study was to investigate the mechanism through which a scoliosis developed in the normal area of the spine, in an animal that is not known to develop a scoliosis spontaneously. We studied the 3D morphology of the compensatory curves that evolved around a traumatically induced coronal plane deformity in a whale. A (dorso-)lateral blunt traumatic injury caused a localized, acute, predominantly lateral deviation of the spine, that subsequently initiated 3D compensatory scoliotic curves in the anatomically normal areas of the spine. These compensatory curves showed rotation of the vertebral bodies into the convexity of the curve, and an apical lordosis (+ 9.4%), which differed significantly from the kyphosis in the spine of the control group (− 2.1%). Although, since the animal was still growing, some wedging of vertebrae occurred, the shape of the vertebral bodies in the sagittal plane showed no difference with the normal, kyphotic shape of the vertebrae in the control group (− 1.8%). The observed lordosis in the compensatory curves of the whale was exclusively located in the intervertebral discs, they showed severe anterior lengthening (+ 99.5%) which was in stark contrast with the kyphotic discs of controls (− 4.6%). This 3D morphology is very similar to what is found in humans with idiopathic scoliosis, but also in human neuromuscular scoliosis as well as in the compensatory curve in human congenital scoliosis^22–25^.

The acute primary scoliosis caused by the traumatic accident, resulted in the head and tail of the whale being out of line and inhibiting proper locomotion and swimming manoeuvres. As many mammals have a vestibular reflex of self-righting^41,42^, the whale most likely compensated this trunk imbalance in an attempt to re align its head to its tail, inducing compensatory curves in the anatomically normal areas of the spine with a 3D morphology that strongly resembles human scoliosis. Whereas most spines in nature require substantial effort to start a permanent rotational deformity due to the stabilizing action of gravity in combination with the trunk’s muscles (i.e. the follower load)^39,43^, the human spine is much less rotationally stable due to its unique sagittal profile with the body’s centre of gravity straight above, rather than in front of the pelvis^13–17,27^. This reduces the stabilizing anterior shear loading and even induces *posteriorly* directed shear loads that render the involved spinal segments unstable in the horizontal plane^1,18,19,28,29,39,44^.

This rare occurrence of scoliosis in a species that is not known to develop a spinal curvature spontaneously, provided a unique chance to study scoliosis in a completely different model. A limitation of this study was that the common minke whale was not compared to non-scoliotic controls of the exact same species. This is due to the low frequency of stranded common minke whales in the Netherlands, in combination with their large size and weight exceeding the capacity of most CT-scanning facilities. However, the smaller harbour porpoise (*Phocoena phocoena*) share strong commonalities in spinal anatomy and were therefore used as controls in the current study^45,46^. Furthermore, the fractured and extensively deformed vertebrae were excluded since proper recognition of the anatomical planes was impossible during CT-scan analysis. However, visual inspection showed wedging mainly in the coronal plane and overview images in the sagittal plane of the CT-scan did not show a significant kyphosis at the site of traumatic injury. Therefore, we could exclude a post-traumatic kyphosis as the initiator of the apical lordosis observed in this study. Also, the observation of a lordosis or kyphosis could be influenced by the fact that the scoliotic whale, nor the controls were alive during CT-scanning and were positioned prone outside of their naturally aquatic habitat. We know from human scoliosis that kyphosis and lordosis are underestimated during prone or supine imaging compared to upright, but there is no difference between prone or supine^47^. Gravity obviously plays an important role in humans, but not in submerged mammals, therefore we feel that the influence of positioning on our results is limited.

The aim of this study was to analyse whether scoliosis can be considered a more generalized compensatory mechanism that occurs independent of cause and/or species. In line with our hypothesis, we observed that the compensatory curves that developed in the normal area of the spine of a whale, that suffered severe but localized trauma to the spine, show strong similarities in 3D configuration with different types of human scoliosis. This suggests a shared and rather uniform mechanical basis, implying that any perceived decompensation of spinal equilibrium can lead to a uniform response, with uniform 3D morphology. The unique biomechanics of the upright human spine^13–17,27^, with significantly decreased rotational stability^1,18,19,39,44^, may explain why only in humans this mechanism can be induced relatively easily, without an obvious cause, and is therefore still called ‘idiopathic’.

## Methods

### Post mortem examination

Since 2008, cetaceans that stranded dead or died shortly after stranding on the Dutch coast are subjected to post mortem examination, which is conducted at the division of pathology of the Faculty of Veterinary Medicine (Utrecht University). The animals described in the current study were not used for scientific or commercial testing. All were free-living whales which died of natural causes or were euthanized on welfare grounds and not for the purpose of this, or other studies. Therefore, since there was no handling of live animals in the current study, according to institutional guidelines, no consent from the Animal Use Committee was required, and animal ethics committee approval was not applicable to this work. On the 8th of July 2019, a young common minke whale washed up on the North Sea beach of Texel, the Netherlands (Fig. 1), and subsequently underwent post mortem investigation aiming to determine the cause of its death. A necropsy and tissue sampling procedure was conducted following internationally standardized guidelines^48^. This included the collection of the following measures: total length (measured from the tip of the rostrum to the fluke notch, in a straight line next to the body, in cm), weight (kg) and blubber thickness. The latter was measured immediately anterior to the dorsal fin at three locations (dorsal, lateral and ventral, in mm). Age class was determined based on total length and gross examination of reproductive organs. Tissue samples from various organs, as well as the vertebral bone, were fixed in 4% phosphate-buffered formalin, embedded in paraffin, cut into 4 µm sections, and stained with haematoxylin and eosin. Samples from vertebra were decalcified prior to paraffin imbedding and staining procedures.

### Diagnostic imaging

Upon gross examination of the whale, the spinal malformation was noted. The entire vertebral column was therefore wrapped in plastic sheets and submitted for computed tomography (CT)-scanning. The spine was positioned in ventral recumbency on the table of a 64-slice sliding gantry CT scanner (Somatom Definition AS, Siemens AG, München, Germany).

### Control group

As common minke whale strandings infrequently occur on the Dutch coast, a control group of the same species was not possible to acquire. Therefore, a control group was assembled of the harbour porpoise; a smaller member of the cetacean family and the most abundant whale species in the North Sea. Harbour porpoises are regularly subjected to post mortem examination and in a previous study focusing on their anatomy, animals were subjected for full-body CT-scan prior to the necropsies^49^. Ten cases which did not present spinal abnormalities and were positioned straight during CT-scanning were selected from this database and used as a control group in this study.

### CT measurements

**Figure 7:**
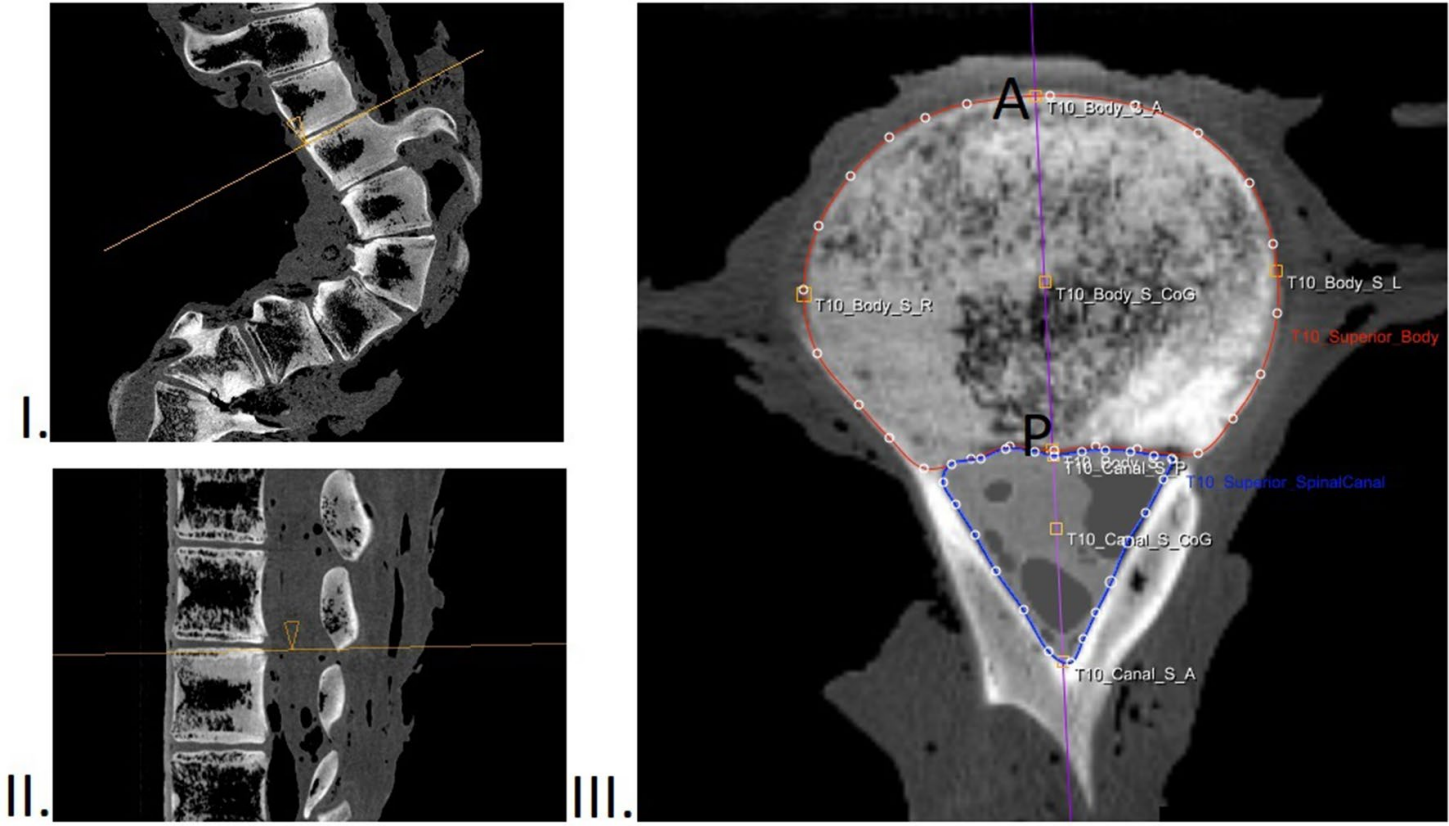
Method of 3D measurements on CT-scans in this study. For all upper and lower endplates, the observer adjusted the plane of view for coronal (**I**) and sagittal (**II**) tilt. In the true transverse plane, the vertebral body and spinal canal were manually segmented (**III**), whereafter the software automatically determined the 3D coordinates of the anterior (A) and posterior (P) point of the endplate, adjusted for rotation and deformity in all planes. The distances between these points were calculated to obtain the anterior and posterior heights of the vertebral bodies and intervertebral discs.

The orientation of the scanned whales in this study was defined the same way as in humans, with anterior indicating the ventral side and posterior indicating the dorsal side, and furthermore cranial, caudal, left and right as standard. The CT-scans of the whale and control group was measured with dedicated software (ScoliosisAnalysis 4.1; Image Sciences Institute, Utrecht, The Netherlands, developed with MeVisLab, MeVis Medical Solutions AG, Bremen, Germany) to measure the direction and amount of rotation, anterior and posterior height of vertebral bodies and vertebral discs in the exact mid-sagittal plane, corrected for deformity in all three planes. This software is in-house developed and validated with excellent intra- and interobserver reliability^50^. This semi-automated method is used and extensively described in multiple earlier studies^23,25,50, 51^. For all upper and lower endplates in the included part of the spine, the observer adjusted the plane of view for coronal and sagittal tilt. In this true transverse plane, the vertebral body and spinal canal were manually segmented by the observer, whereafter the software automatically determined the 3D coordinates of the anterior and posterior point of the endplate, adjusted for rotation and deformity in all planes. The distances between these points were calculated to obtain the anterior and posterior heights of the vertebral bodies and intervertebral discs (Fig. 7). This was done for all the compensatory curves (Cobb-to-Cobb). The corresponding levels of the spine analysed in the whale were also measured in controls. After measurements, the anterior–posterior length discrepancy (AP%) was calculated as [(anterior length – posterior length)/(posterior length)] × 100%, for the total compensatory curved spine, and for the vertebral bodies and the intervertebral discs separately. Endplates severely affected by trauma or wedging were excluded, as proper segmentation was not possible. Positive AP% values indicated that the anterior (ventral) side was longer than the posterior (dorsal) side.

### Statistical analysis

The mean AP% results for the total curve, the vertebral bodies and the intervertebral bodies were determined for the minke whale and for the non-scoliotic harbour porpoise control group given with ± standard deviation. The differences in mean AP% between the scoliotic whale and non-scoliotic controls were tested with an independent samples T-test. The data for Figs. 5, 6 were provided separately (Supplementary Information). Statistical analysis was performed in SPSS 25.0 for Windows (IBM, Armonk, NY, USA). The level of statistical significance was set at p ≤ 0.05.

### Animal ethics committee approval

The animals described in the current study were not used for scientific or commercial testing. All were free-living whales which died of natural causes or were euthanized on welfare grounds and not for the purpose of this, or other studies. Therefore, since there was no handling of live animals in the current study, according to institutional guidelines, no consent from the Animal Use Committee was required, and animal ethics committee approval was not applicable to this work.

## Supplementary Information

Supplementary Information.

## Data Availability

All generated or analysed data in this study are included in this article or the supplementary information files.
